# The global catalogue of microorganisms 10K type strain sequencing project: closing the genomic gaps for the validly published prokaryotic and fungi species

**DOI:** 10.1093/gigascience/giy026

**Published:** 2018-03-22

**Authors:** Linhuan Wu, Kevin McCluskey, Philippe Desmeth, Shuangjiang Liu, Sugawara Hideaki, Ye Yin, Ohkuma Moriya, Takashi Itoh, Cha Young Kim, Jung-Sook Lee, Yuguang Zhou, Hiroko Kawasaki, Manzour Hernando Hazbón, Vincent Robert, Teun Boekhout, Nelson Lima, Lyudmila Evtushenko, Kyria Boundy-Mills, Boyke Bunk, Edward R B Moore, Lily Eurwilaichitr, Supawadee Ingsriswang, Heena Shah, Su Yao, Tao Jin, Jinqun Huang, Wenyu Shi, Qinglan Sun, Guomei Fan, Wei Li, Xian Li, İpek Kurtböke, Juncai Ma

**Affiliations:** 1 Microbial Resource and Big Data Center, Institute of Microbiology, Chinese Academy of Sciences, Beijing 100101, China; 2 State Key Laboratory of Microbial Resources, Institute of Microbiology, Chinese Academy of Sciences, Beijing 100101, China; 3 WFCC-MIRCEN World Data Center for Microorganisms, Beijing 100101, China; 4 World Federation of Culture Collections (WFCC); 5 Fungal Genetics Stock Center, Kansas State University, Manhattan, KS 66506, USA; 6 Belgian Coordinated Collections of Micro-organisms Program, Belgian Science Policy Office, Brussels 231 1050, Belgium; 7 National Institute of Genetics, Yata, Mishima 411-8540, Japan; 8 BGI Genomics, BGI-Shenzhen, Shenzhen 518083, China; 9 Japan Collection of Microorganisms/Microbe Division, RIKEN BioResource Center, Koyadai 3-1-1, Tsukuba, Ibaraki 305-0074, Japan; 10 Korean Collection for Type Cultures, Korea Research Institute of Bioscience and Biotechnology (KRIBB), 181 Ipsin-gil, Jeongeup-si, Jeollabuk-do, 56212, Republic of Korea; 11 China General Microbiological Culture Collection Center, Institute of Microbiology, Chinese Academy of Sciences, Beijing10010, China; 12 NITE Biological Resource Center, National Institute of Technology and Evaluation, 2-5-8 Kazusakamatari, Kisarazu, Chiba 292-0818, Japan; 13 American Type Culture Collection, 10801 University Boulevard, Manassas, VA 20110, USA; 14 Westerdijk Fungal Biodiversity Institue, Utrecht 3534CT, Netherlands; 15 Institute of Biodiversity and Ecosystem Dynamics, University of Amsterdam, Spui 21 1012 WX Amsterdam, Netherlands; 16 Shanghai Key Laboratory of Molecular Medical Mycology, Shanghai Institute of Mycology, Shanghai Changzheng Hospital, Shanghai 200003, China; 17 Micoteca da Universidade do Minho, Biological Engineering Centre, 4710-057 Braga, Portugal; 18 All-Russian Collection of Microorganisms, GK Skryabin Institute of Biochemistry and Physiology of Microorganisms RAS, Pushchino, Moscow Region 142290, Russia; 19 Phaff Yeast Culture Collection, Food Science and Technology Department, University of California Davis, 1 Shields Avenue, Davis, CA 95616–8598, USA; 20 Leibniz-Institute DSMZ – German Collection of Microorganisms and Cell Cultures, D-38124 Braunschweig, Germany; 21 Culture Collection University of Gothenburg (CCUG), Sahlgrenska Academy of the University of Gothenburg, SE-41346 Gothenburg, Sweden; 22 Bioresources Technology Unit, Thailand Bioresource Research Center, National Center for Genetic Engineering and Biotechnology, Bangkok National Science and Technology Development Agency, 113, Thailand; 23 National Collection of Type Cultures, Public Health England, Porton Down, Salisbury, Wiltshire SP4 0JG, UK; 24 China Center of Industrial Culture Collection, Beijing 100015, China; 25 Faculty of Science, Health, Education and Engineering, University of the Sunshine Coast, Maroochydore, Queensland 4558, Australia

**Keywords:** phylogenomics, taxonomy, biodiversity, whole-genome sequencing, type strains, bacteria, Archaea, fungi

## Abstract

Genomic information is essential for taxonomic, phylogenetic, and functional studies to comprehensively decipher the characteristics of microorganisms, to explore microbiomes through metagenomics, and to answer fundamental questions of nature and human life. However, large gaps remain in the available genomic sequencing information published for bacterial and archaeal species, and the gaps are even larger for fungal type strains. The Global Catalogue of Microorganisms (GCM) leads an internationally coordinated effort to sequence type strains and close gaps in the genomic maps of microorganisms. Hence, the GCM aims to promote research by deep-mining genomic data.

## Introduction

Microorganisms are the most abundant organisms on Earth. The total diversity of prokaryotes may comprise up to 10^9^ species [1]. For prokaryotic species, only new names published in the *International Journal of Systematic andEvolutionary Microbiology* (IJSEM) as an original article or in the “Validation Lists” are considered valid. As of the end of 2017, 15,081 valid prokaryotic species and subspecies were published compared to 12,981 at the end of December 2015 [2]. The number of publications increased by 1,088 in 2016 and by 1,012 in 2017. The most commonly accepted estimate of the number of existing fungal species is 2.27 million, as hypothesized by Hawksworth [3], while the number of species reported in the *Dictionary of Fungi* is only about 100,000.

The taxonomy of microorganisms, including their classification, identification, and nomenclature, has developed from morphological and metabolic characterization to incorporate numerical taxonomy based on phenetic analyses, chemotaxonomy, and finally polyphasic approaches that combine phenotypic, chemotaxonomic, genotypic, and genomic information. The IJSEM recently announced that since January 2018, authors of new taxa descriptions have been asked to provide genome sequence data with descriptions of novel taxa with their manuscript submissions [4]. As such, the taxonomy of microorganisms has entered the genomics era. A genomic “gold standard” for consistent microbial species definitions is urgently needed. To meet this end, a fundamental step is to sequence the type strains of validly published prokaryotic and fungal species.

In addition to the microorganisms that can be isolated and maintained *in situ*, the vast majority of microorganisms cannot yet be cultivated and thus are relatively poorly studied. Culture-independent approaches have been developed to investigate the compositions and functions of environmental and human microbiomes. However, accurate taxonomic and functional predictions based on metagenomic data are dependent on the availability of high-quality reference genomic data [5]. Therefore, sequencing the genomes of type strains of recognized microbial species will provide a taxonomic context for metagenomic data analysis, which is commonly comprised of short and incomplete sequences from complex environmental communities.

Microorganisms possess extensive genomic and metabolic diversity, which makes them ideal biotechnological tools. Decoding the full genomes of the type strains of various species in order to provide reference genomes will thus enable genes to be associated with functions, such as metabolic activity, virulence, antibiotic production or resistance, biomass deconstruction, cellulose agricultural nitrogen fixation, and the liberation of environmental phosphorus. Access to microbial genomic sequences will significantly contribute to future studies in microbial biology, ecology, and biochemistry and these will, in turn, accelerate the discovery of new natural products and drugs [6].

## The Current Status of the Strain Sequencing Project

Descriptions of prokaryotic species are based on living cultures, and one representative strain is designated as the nomenclatural “type.” The IJSEM and the International Committee on Systematics of Prokaryotes require that the type strains of new species be deposited in at least two recognized collections in two countries. The type strains of 15,081 prokaryotic species are widely preserved in more than 130 culture collections. In mycology the trend is similar, although hitherto a fungal type specimen must be metabolically inactive.

Presently, the selection of strains for whole-genome sequencing is based predominantly on medical, ecological, or industrial importance, which often leads to bias in assessing phylogenetic relationships. There are several ongoing phylogenetic-based microbial sequencing projects. The Genomic Encyclopedia of Bacteria and Archaea (GEBA), led by the US Department of Energy Joint Genome Institute (US DOE JGI), has pioneered the partnership between culture collections and sequencing projects. The GEBA project published 1,003 whole-genome sequences of type strains in 2017 as the outcome of its first stage [7]. GEBA started the new stage of the project in 2015, which has a focus on the genomes of soil, plant-associated, and newly described type strains [8].

Similarly, the US DOE JGI, in collaboration with international research teams, conducted a 5-year project to sequence 1,000 fungal genomes from across the Fungal Tree of Life [9]. The overall plan is to fill in gaps in the Fungal Tree of Life by sequencing at least two reference genomes from the more than 500 recognized families of fungi.

Many type strains of microbial species remain unsequenced. Hence, the World Federation of Culture Collections (WFCC) and the World Data Centre for Microorganisms (WDCM) have initiated an international community-led project to sequence the full genomes of microbial type strains to support continued scientific discovery and biotechnological utilization. Considering the wide distribution of type strains, cooperation across the global culture collection community is essential for success.

## Emerging Enhancements of Culture Collection in the Genomic Era

Efforts made by culture collection curators to explore the diversity of microorganisms and to harness their genes, properties, and products remain insufficient. While type collections are not always large or diverse, the genome sequencing efforts of the Global Catalogue of Microorganisms (GCM) will increase access to resources in smaller collections.

The WDCM is the data center of the WFCC and the Microbial Resources Centers Network. The WDCM is working on facilitating the application of cutting-edge information technology to improve the interoperability of microbial data, promote access and use of data, and coordinate international cooperation among culture collections, scientists, and other user communities. The first stage of the GCM project, started in 2012, focused on sharing strain catalogue data from culture collections [10]. The proposed type strain sequencing project is the continuation of the GCM project as its second stage, GCM 2.0.

## Project Development and Current Progress

The project was first announced during the 14th International Culture Collections Conference, held in Singapore in July 2017 in conjunction with the International Union of Microbiological Societies (IUMS) conferences. Following that, in October 2017, a ceremony was held in Beijing, China, to launch the project, at which representatives from the following culture collections were present: the American Type Culture Collection (ATCC), Belgian Coordinated Collections of Microorganisms (BCCM), Biological Resource Center, Japan National Institute of Technology and Evaluation (NBRC), Culture Collection of the University of Gothenburg (CCUG), China General Microbiological Culture Collection Center (CGMCC), Fungal Genetics Stock Center (Kansas State University, USA) (FGSC), Japan Collection of Microorganisms (JCM), Korean Collection for Type Cultures (KCTC), Leibniz-Institut DSMZ-Deutsche Sammlung von Mikroorganismen und Zellkulturen (DSMZ), Micoteca da Universidade do Minho (Portugal) (MUM), Thailand Bioresource Research Center (TBRC), and Westerdijk Fungal Biodiversity Institute (CBS). The meeting representatives held detailed discussions on the organization and operational procedures of the GCM 2.0 project. Some culture collections (e.g., All-Russian Collection of Microorganisms [VKM], China Center of Industrial Culture Collection [CICC], Phaff Yeast Culture Collection [University of California Davis, USA;(UCD-FST], Thailand Institute of Scientific and Technological Research Culture Collection [TISTR], and UK National Collection of Type Cultures [NCTC]) did not have a representative present at the launch ceremony but expressed their willingness to join the collaboration.

It is expected that the project will be completed within 5 years, including a pilot stage in the first year. GCM 2.0 includes two core subprojects, sequencing 10,000 bacterial and archaeal type strains and sequencing of some of the fungal type strains. It will also embrace several satellite projects on specific scientific targets. GCM 2.0 is coordinated by a steering committee and five interlinked working groups: Bacteria Selection, Fungi Selection, Standard Operational Procedures, Databases, and Intellectual Property Rights and Legal Issues.

The project has established standard operational procedures for DNA extraction, sample submission, sequencing, and data processing to ensure that all genetic resources, data, and metadata associated with type strains are appropriately obtained, recorded, and stored. A project proposed by the WDCM, “AWI 20170: Specification on Data Integration and Publication in Microbial Resource Centers,” which would meet International Organization for Standardization standards, is under development. The raw data and annotation results generated from this project will be published on the GCM portal. Following norms established for genome projects coming from the Bermuda Principles and Fort Lauderdale agreement, the resulting genomic data will also be made freely available in public databases, including those maintained by the National Center for Biotechnology Information, the European Molecular Biology Laboratory, and the DNA Data Bank of Japan, after completion of data analysis and annotation and ensurance that the data has met a set of quality criteria.

All validly published bacterial and archaeal type strains, as well as selected reference fungal type strains that are frequently used for functional or phylogenetic studies, will be on the list of candidates for sequencing. Each strain has documentation issued by the providing culture collection, which ensures the purity and identity of the type strain. BGI-Shenzhen will support the microbial genome sequencing and assist with the data analysis for this project. Sampling works for the pilot stage have been initiated. The project has established a global network to collect approximately 800 candidate type strain samples from American, British, Belgian, Chinese, Dutch, Japanese, Korean, Portuguese, Russian, Swedish, and Thai collections. Although extracted DNA samples are much preferred, it is also acceptable to send cultured cell samples of the strains. Importantly, under the terms of Nagoya Protocol, GCM 2.0 will respect the access and benefit-sharing regulations of all countries.

The GCM type strain sequencing project encourages all culture collections to participate in this international collaborative project. Interested parties should be willing to provide DNA for type strains held in their collections. All microbiologists and institutions from related fields are welcome to submit subprojects for genomic data-related research questions. Brief proposals, including questions to be addressed and type strains to be sequenced and analyzed, should be emailed to Dr Juncai Ma at ma@im.ac.cn. Once a proposal is granted as a subproject, the scientist(s) will be asked to lead the full genome analysis and jointly publish the generated outcomes.

## Conclusion

As a collaborative network of international culture collections, GCM 2.0 will contribute to a genome-based microbial taxonomic framework, establishing high-quality complete genome sequences as the new gold standard. The resulting knowledge and tools generated through this project will not only directly facilitate the identification of microorganisms but will also improve our ability to predict new gene complexes and their functions from microbial communities. Thus, our knowledge of the hitherto undiscovered microbial diversity will be expanded, which may lead to the sustainable utilization of microbial resources for human benefit.

## Abbreviations

GEBA, Genomic Encyclopedia of Bacteria and Archaea; GCM, Global Catalogue of Microorganisms; IJSEM,*International Journal of Systematic and Evolutionary Microbiology*; US DOE JGI, Uniteed States Department of Energy Joint Genome Institute; WDCM, World Data Centre for Microorganisms; WFCC, World Federation of Culture Collections.

## Supplementary Material

GIGA-D-18-00079_Original_Submission.pdf

GIGA-D-18-00079_Revision_1.pdf

Response_to_Reviewer_Comments_Original_Submission.pdf

## References

[1] Curtis TP, Sloan WT, Scannell JW Estimating prokaryotic diversity and its limits. Proc Natl Acad Sci. 2002;99:10494–9. 12097644 10.1073/pnas.142680199 PMC124953

[2] Garrity GM A new genomics-driven taxonomy of bacteria and archaea: are we there yet? J Clin Microbiol. 2016;54:1956–63. 27194687 10.1128/JCM.00200-16 PMC4963521

[3] Hawksworth DL The magnitude of fungal diversity: the 1.5 million species estimate revisited. Mycol Res. 2001;105:1422–32.

[4] Chun J, Oren A, Ventosa A, Proposed minimal standards for the use of genome data for the taxonomy of prokaryotes. Int J Syst Evol Microbiol. 2018;68:461–6. 29292687 10.1099/ijsem.0.002516

[5] Kim Y, Koh I, Rho M Deciphering the human microbiome using next-generation sequencing data and bioinformatics approaches. Methods. 2015;79–80:52–59. 10.1016/j.ymeth.2014.10.022 25448477

[6] Kang HS Phylogeny-guided (meta)genome mining approach for the targeted discovery of new microbial natural products. J Ind Microbiol Biotechnol. 2017;44:285–93. 27885438 10.1007/s10295-016-1874-z

[7] Mukherjee S, Seshadri R, Varghese NJ 1,003 reference genomes of bacterial and archaeal isolates expand coverage of the Tree of Life. Nat Biotechnol. 2017;35:676–83. 28604660 10.1038/nbt.3886

[8] Whitman WB, Woyke T, Klenk HP Genomic encyclopedia of bacterial and archaeal type strains, phase III: the genomes of soil and plant-associated and newly described type strains. Stand in Genomic Sci. 2015;10:26. 26203337 10.1186/s40793-015-0017-x PMC4511459

[9] 1000 fungal genomes Project. https://jgi.doe.gov/our-science/science-programs/fungal-genomics/1000-fungal-genomes/ Accessed March 1, 2017.

[10] Wu L, Sun Q, Desmeth P World data centre for microorganisms: an information infrastructure to explore and utilize preserved microbial strains worldwide. Nucleic Acids Res. 2017;45:D611–8. 28053166 10.1093/nar/gkw903 PMC5210620

