# Supplementary material for: The global catalogue of microorganisms 10K type strain sequencing project: closing the genomic gaps for the validly published prokaryotic and fungi species
Source: Gigascience. 2018 Mar 22;7(5):giy026. doi: 10.1093/gigascience/giy026 (PMC5941136; doi:10.1093/gigascience/giy026)
Supplement: GIGA-D-18-00079_Original_Submission.pdf [file giy026_giga-d-18-00079_original_submission.pdf]

# GigaScience

## The Global Catalogue of Microorganisms 10K type strain sequencing project: An international effort of the culture collection community toward closing the genomic gaps for the validly published prokaryotic and fungi species

--Manuscript Draft--

| | |
|---|---|
| **Manuscript Number:** | GIGA-D-18-00079 |
| **Full Title:** | The Global Catalogue of Microorganisms 10K type strain sequencing project: An international effort of the culture collection community toward closing the genomic gaps for the validly published prokaryotic and fungi species |
| **Article Type:** | Commentary |
| **Funding Information:** | Strategic Priority Research Program of the Chinese Academy of Sciences (XDA19050301) — Dr. Juncai Ma<br>Bureau of International Cooperation, Chinese Academy of Sciences (153211KYSB20160029) — Dr. Juncai Ma<br>Bureau of International Cooperation, Chinese Academy of Sciences (153211KYSB20150010) — Dr. Juncai Ma<br>National key Research Program of China (2017YFC1201202) — Dr. Juncai Ma<br>National key Research Program of China (2016YFC1201303) — Dr. Juncai Ma<br>National key Research Program of China (2016YFC0901702) — Dr. Juncai Ma<br>13th Five-year Informatization Plan of Chinese Academy of Sciences (XXH13506) — Dr. Juncai Ma<br>National Science Foundation for Young Scientists of China (31701157) — Dr. Linhuan Wu |
| **Abstract:** | Genomic information is essential to underpin taxonomic, phylogenetic and functional studies to comprehensively decipher the characteristics of microorganisms, to explore microbiomes through metagenomics and also answer fundamental questions of nature and human life. However, there is still a big gap of genomic sequencing information for the bacterial and archaeal species with validly published names. For fungal type strains, the remaining gap is even larger. Global Catalogue of Microorganisms (GCM)'s led type strain sequencing project is an international coordinated effort to close current gaps in the genomic maps of microbes and hence to promote research through deep-mining of the genomic data. |
| **Corresponding Author:** | Juncai Ma<br><br>CHINA |
| **Corresponding Author Secondary Information:** | |
| **Corresponding Author's Institution:** | |
| **Corresponding Author's Secondary Institution:** | |
| **First Author:** | Linhuan Wu |
| **First Author Secondary Information:** | |
| **Order of Authors:** | Linhuan Wu<br>Kevin McCluskey<br>Philippe Desmeth |

Powered by Editorial Manager® and ProduXion Manager® from Aries Systems Corporation

| | |
|---|---|
| | Shuangjiang Liu |
| | Sugawara Hideaki |
| | Ye Yin |
| | Ohkuma Moriya |
| | Takashi Itoh |
| | Cha Young Kim |
| | Jung-Sook Lee |
| | Yuguang Zhou |
| | Hiroko Kawasaki |
| | Manzour Hazbón |
| | Vincent Robert |
| | Teun Boekhout |
| | Nelson Lima |
| | Lyudmila Evtushenko |
| | Kyria Boundy-Mills |
| | Boyke Bunk |
| | Edward R.B. Moore |
| | Lily Eurwilaichitr |
| | Supawadee Ingsriswang |
| | Heena Shah |
| | Su Yao |
| | Tao Jin |
| | Jinqun Huang |
| | Wenyu Shi |
| | Qinglan Sun |
| | Guomei Fan |
| | Wei Li |
| | Xian Li |
| | Ipek Kurtböke |
| | Juncai Ma |
| **Order of Authors Secondary Information:** | |
| **Opposed Reviewers:** | |
| **Additional Information:** | |
| **Question** | **Response** |
| Are you submitting this manuscript to a special series or article collection? | No |
| **Experimental design and statistics**<br><br>Full details of the experimental design and statistical methods used should be given in the Methods section, as detailed in our | Yes |

| | |
|---|---|
| Minimum Standards Reporting Checklist. Information essential to interpreting the data presented should be made available in the figure legends.<br><br>Have you included all the information requested in your manuscript? | |
| **Resources**<br><br>A description of all resources used, including antibodies, cell lines, animals and software tools, with enough information to allow them to be uniquely identified, should be included in the Methods section. Authors are strongly encouraged to cite Research Resource Identifiers (RRIDs) for antibodies, model organisms and tools, where possible.<br><br>Have you included the information requested as detailed in our Minimum Standards Reporting Checklist? | Yes |
| **Availability of data and materials**<br><br>All datasets and code on which the conclusions of the paper rely must be either included in your submission or deposited in publicly available repositories (where available and ethically appropriate), referencing such data using a unique identifier in the references and in the "Availability of Data and Materials" section of your manuscript.<br><br>Have you have met the above requirement as detailed in our Minimum Standards Reporting Checklist? | Yes |

Manuscript

Click here to download Manuscript GCM 10K type strain sequencing project -submission.docx

# The Global Catalogue of Microorganisms 10K type strain sequencing project: An international effort of the culture collection community toward closing the genomic gaps for the validly published prokaryotic and fungi species

Linhuan Wu[1,2,3], Kevin McCluskey[4,23], Philippe Desmeth[5,23], Shuangjiang Liu[2,23], Sugawara Hideaki[6], Ye Yin[7], Ohkuma Moriya[8], Takashi Itoh[8], Cha Young Kim[9], Jung-Sook Lee[9], Yuguang Zhou[10], Hiroko Kawasaki[11], Manzour Hernando Hazbón[12], Vincent Robert[13], Teun Boekhout[13,24,25], Nelson Lima[14], Lyudmila Evtushenko[15], Kyria Boundy-Mills[16,23], Boyke Bunk[17], Edward R.B. Moore[18], Lily Eurwilaichitr[19,23] Supawadee Ingsriswang[19], Heena Shah[20], Su Yao[21], Tao Jin[7], Jinqun Huang[7], Wenyu Shi[1], Qinglan Sun[1], Guomei Fan[1], Wei Li[1], Xian Li[1], İpek Kurtböke[22,23 *] & Juncai Ma[1,2,3,23*]

#To whom correspondence should be addressed: İpek Kurtböke IKurtbok@usc.edu.au & Juncai Ma ma@im.ac.cn

1 Microbial Resource and Big Data Center, Institute of Microbiology, Chinese Academy of Sciences, Beijing 100101, China

2 State Key Laboratory of Microbial Resources, Institute of Microbiology, Chinese Academy of Sciences, Beijing 100101, China

3 WFCC-MIRCEN World Data Center for microorganisms, WDCM, Beijing 100101, China

4 Fungal Genetics Stock Center, Kansas State University, Manhattan KS 66506, USA

5 Belgian Coordinated Collections of Micro-organisms Programme, Belgian, Science Policy Office, Brussels 231 1050, Belgium

6 National Institute of Genetics, Yata, Mishima 411-8540, Japan

7 BGI Genomics, BGI-Shenzhen, Shenzhen 518083, China

8 Japan Collection of Microorganisms/ Microbe Division, RIKEN BioResource Center, Koyadai 3-1-1, Tsukuba, Ibaraki 305-0074, Japan

9 Korean Collection for Type Cultures (KCTC), Korea Research Institute of Bioscience and 30 Biotechnology (KRIBB), 181 Ipsin-gil, Jeongeup-si, Jeollabuk-do 56212, Republic of Korea

10 China General Microbiological Culture Collection Center, Institute of Microbiology, Chinese Academy of Sciences, Beijing10010, China

11 NITE Biological Resource Center (NBRC), National Institute of Technology and 24 Evaluation, 2-5-8 Kazusakamatari, Kisarazu, Chiba 292-0818, Japan

12 American Type Culture Collection, 10801 University Boulevard, Manassas, VA 20110, USA

13 Westerdijk Fungal Biodiversity Institue, Utrecht 3534CT, Netherlands

14 Micoteca da Universidade do Minho, Biological Engineering Centre, 4710-057 Braga, Portugal

15 All-Russian Collection of Microorganisms, G.K. Skryabin Institute of Biochemistry and Physiology of Microorganisms RAS, Pushchino, Moscow region 142290, Russia

16 Phaff Yeast Culture Collection Food Science & Technology Department University of California Davis One Shields Avenue, Davis, CA 95616-8598, USA

17 Leibniz-Institute DSMZ – German Collection of Microorganisms and Cell Cultures, D-38124 Braunschweig, Germany

18 Culture Collection University of Gothenburg (CCUG), Sahlgrenska Academy of the University of Gothenburg, SE-41346 Gothenburg, Sweden

19 Bioresources Technology Unit Thailand Bioresource Research Center (TBRC), National Center for Genetic Engineering and Biotechnology (BIOTEC), Bangkok National Science and Technology Development Agency (NSTDA), 113, Thailand

20 National Collection of Type Cultures (NCTC), Public Health England (PHE), Porton Down,Salisbury,Wiltshire,SP4 0JG, UK

21 China Center of Industrial Culture Collection (CICC), Beijing 100015, China

22 Faculty of Science, Health, Education and Engineering University of the Sunshine Coast Maroochydore DC Queensland 4558, Australia

23 World Federation of Culture Collections (WFCC)

24 Institute of Biodiversity and Ecosystem Dynamics (IBED), University of Amsterdam, Spui 21 1012 WX Amsterdam, The Netherlands

25 Shanghai Key Laboratory of Molecular Medical Mycology, Shanghai Institute of Mycology, Shanghai Changzheng Hospital, Shanghai 200003, China

Email address of the authors:

Linhuan Wu: wulh@im.ac.cn

Kevin McCluskey: mccluskeyk@ksu.edu

Philippe Desmeth: Philippe.DESMETH@belspo.be

Shuangjiang Liu: liusj@im.ac.cn

Sugawara Hideaki: hsugawar@nig.ac.jp

Ye Yin: yinye@genomics.cn

Ohkuma Moriya: mohkuma@riken.jp

Takashi Itoh: ito@jcm.riken.jp

Cha Young Kim: kimcy@kribb.re.kr

Jung-Sook Lee: jslee@kribb.re.kr

Yuguang Zhou: zhouyg@im.ac.cn

Hiroko Kawasaki: kawasaki-hiroko@nite.go.jp

Manzour Hernando Hazbón: mhazbon@atcc.org

Vincent Robert: v.robert@westerdijkinstitute.nl

Teun Boekhout: t.boekhout@westerdijkinstitute.nl

Nelson Lima: nelson@ie.uminho.pt

Lyudmila Evtushenko: lie99@mail.ru

Kyria Boundy-Mills: klbmills@ucdavis.edu

Boyke Bunk: boyke.bunk@dsmz.de

Edward R.B. Moore: erbmoore@ccug.se

Lily Eurwilaichitr: lily@biotec.or.th

Supawadee Ingsriswang: supawadee@biotec.or.th

Heena Shah: Heena.Shah@phe.gov.uk

Su Yao: milly@china-cicc.org

Tao Jin: jintao@genomics.cn

Jinqun Huang: huangjinqun@genomics.cn

Wenyu Shi: shiwy@im.ac.cn

Qinglan Sun: sunql@im.ac.cn

Guomei Fan: gmfan@im.ac.cn

Wei Li: liwei2227@im.ac.cn

Xian Li: lixian@im.ac.cn

İpek Kurtböke: IKurtbok@usc.edu.au

Juncai Ma: ma@im.ac.cn

## Abstract

Genomic information is essential to underpin taxonomic, phylogenetic and functional studies to comprehensively decipher the characteristics of microorganisms, to explore microbiomes through metagenomics and also answer fundamental questions of nature and human life. However, there is still a big gap of genomic sequencing information for the bacterial and archaeal species with validly published names. For fungal type strains, the remaining gap is even larger. Global Catalogue of Microorganisms (GCM)'s led type strain sequencing project is an international coordinated effort to close current gaps in the genomic maps of microbes and hence to promote research through deep-mining of the genomic data.

**Keywords:** Phylogenomics, Taxonomy, Biodiversity, Whole-genome sequencing, Type strains, Bacteria, Archaea, Fungi

## Background

Microorganisms are the most abundant organisms on the earth. The total prokaryotic diversity may comprise up to $10^9$ species[1]. For prokaryotic species, only new names published in the International Journal of Systematic and Evolutionary Microbiology (IJSEM) as an original article or in the 'Validation Lists' are considered to be valid. At the end of 2017, the number of validly published species and subspecies of prokaryotes was 15,081 while that was 12,981 at the end of December 2015[2], increasing by 1088 and 1012 respectively in the year of 2016 and 2017. The most commonly accepted estimation on the number of existing fungal species is the 2.27 million species hypothesized by Hawksworth[3] while the number of species reported in the Dictionary of Fungi is only about 100,000.

The taxonomy of microorganisms, including classification, identification and nomenclature, has developed from morphological and metabolic characterization, numerical taxonomy based on phenetic analyses, then incorporated chemotaxonomy, and finally to polyphasic approaches combining phenotypic, chemotaxonomic, genotypic and genomic information. The IJSEM recently announced that starting January 2018, authors of new taxa descriptions are asked to provide genome sequence data with descriptions of novel taxa for manuscript submission[4]. As such, taxonomy of microorganisms thus entered into the genomics era. A genomic 'gold standard' for setting a consistent microbial species definition are urgently needed. To meet this end, sequencing the Type strains of the validly published prokaryotic and fungal species is

a foundational step.

Besides the microorganisms that can be isolated and maintained *in situ,* the vast majority of microorganisms are not yet cultivated, and thus are relatively poorly studied. Culture-independent approaches have been developed to investigate the compositions and functions of the environmental and human microbiomes. However, accurate taxonomic and functional predictions based upon metagenomic data are dependent upon the availability of high-quality reference genomic data[5]. Therefore, genome sequencing of the Type strains of recognized microbial species will provide a taxonomic context for metagenomic data analysis, which is commonly comprised by short and incomplete sequences from complex environmental communities.

Microorganisms, possessing extensive genomic and metabolic diversity are ideal tools for biotechnology. Decoding the full genomes of reference Type strains of species will thus enable association of gene content with function, such as metabolic activity, virulence, antibiotic production or resistance, biomass deconstruction, cellulose agricultural nitrogen fixation and the liberation of environmental phosphorus, etc. Access to microbial genomic sequences will significantly contribute to future studies of microbial biology, ecology, and biochemistry and these will, in turn, accelerate the discovery of new natural products and drugs [5].

## Current status of strain sequencing project

The descriptions of prokaryotic species are based on living cultures and one representative strain is designated as the nomenclatural “type”. It is a requirement of IJSEM and the International Committee on Systematics of Prokaryotes (ICSP) that the

Type strains of new species should be deposited in, at least, two recognized collections in two different countries. The Type strains of 15,081 prokaryotic species are widely preserved in more than 130 culture collections. In mycology the trend is similar, although hitherto a fungal type specimen has to be metabolically inactive.

At present, the selection of strains for whole-genome sequencing has been based predominantly on medical, ecological or industrial importance, which, often leads to bias in assessing phylogenetic relationships. There are several ongoing phylogenetic based microbial sequencing projects. The Genomic Encyclopedia of Bacteria and Archaea (GEBA) led by the US Department of Energy Joint Genome Institute (US DOE JGI) has pioneered the partnership between culture collections and sequencing projects. The GEBA project has published the outcomes of its first stage 1,003[7] Type strain whole-genome sequences at year 2017. The newly started third-stage of GEBA has a focus on the genomes of soil, plant-associated, and newly described Type strains[8].

Similarly, the US DOE JGI collaborated with international research teams conducted a five-year project to sequence 1000 fungal genomes from across the Fungal Tree of Life[9]. The overall plan is to fill in gaps in the Fungal Tree of Life by sequencing at least two reference genomes from the more than 500 recognized families of fungi.

A large number of microbial species Type strains remain to un-sequenced. Hence, the World Federation of Culture Collections (WFCC) and the World Data Centre for Microorganisms (WDCM) have initiated an international community-led project towards full genome sequencing of microbial Type strains to support the continued scientific discovery and biotechnology utilization. Considering the wide distribution of

Type strains, international cooperation of the global culture collection community is essential for success.

## Emerging enhancements of culture collection in the genomic era

Current efforts from culture collections to explore the diversity of microorganisms and to harness their genes, properties and products are still insufficient. While type collections are not always large and diverse, the genome sequence efforts of GCM will obviously make the resources in smaller collections more accessible.

The World Data Center for Microorganisms (WDCM) is the data center of the World Federation for Culture Collections (WFCC) and the Microbial Resources Centers Network (MIRCEN). The WDCM is working on facilitating the application of cutting-edge information technology to improve the interoperability of microbial data, to promote access and use of data, and coordinate international cooperation between culture collections, scientists and other user communities. The first stage of the Global Catalogue of Microorganisms (GCM) project started in 2012 which focused on the data sharing of strain catalogue from culture collections[10]. The proposed Type strain sequencing project is the continuation of the GCM project as its second stage, GCM 2.0.

## Project development and current progress

The project was firstly announced during the 14$^{th}$ International Culture Collections Conference held in July 2017 in Singapore in conjunction with the IUMS conferences. After that, a launching ceremony in Beijing was held in October 2017. Culture collections including ATCC, BCCM, CBS, CCUG, CGMCC, DSMZ, FGSC, JCM,

KCTC, MUM NBRC, and TBRC sent representatives to join the launching ceremony and had thorough discussions on the organization and operational procedures of the GCM 2.0 project. Some culture collections, such as CICC, NCTC, TISTR, UCD-FST, and VKM, although not present in the launching ceremony, expressed their willingness to join the cooperation.

The entire project is expected to be completed in 5 years, including a pilot stage in the first year. The GCM 2.0 includes two core subprojects, the 10K bacterial and archaeal Type strain sequencing and the fungal Type strain sequencing. It will also embrace several satellite projects based on specific scientific targets. GCM 2.0 coordinates its projects through a steering committee and five interlinked working groups: 1) Bacteria Selection, 2) Fungi Selection, 3) Standard Operational Procedures (SOPs), 4) Database and, 5) Intellectual Property Right and Legal Issue.

The project has established SOPs for DNA extraction, sample submission, sequencing and data processing to ensure that all the genetic resources, data and associated metadata of Type strains are appropriately obtained, recorded and stored. A project of ISO standard, proposed by the WDCM, AWI 20170: Specification on data integration and publication in microbial resource centres is under development. Data generated from this project will be published on the GCM portal and also deposited in public databases, NCBI/EMBL/DDBJ and others.

All validly published bacterial and archaeal type strains, as well as selected reference fungal type strains frequently used for functional or phylogenetic studies will be on the candidate sequencing list. Each of these strains has the documentation issued by the

providing culture collection, which assures purity and identity as the Type strain. Beijing Genomics Institute (BGI) will support the microbial genome sequencing and part of data analysis for this project. Sampling works for the pilot-stage have been initiated. The project has established a global network to collect approximately 800 candidate Type strain samples of collections from Belgium, China, Japan, Korea, The Netherlands, Portugal, Russia, Sweden, Thailand, USA, and UK. Although extracted DNA samples are highly preferred, sending the cultured cell samples of the strains is also acceptable. Importantly, GCM 2.0 will respect all countries regulations regarding Access and Benefit-Sharing (ABS) under Nagoya Protocol.

The GCM Type strain sequencing project encourages all culture collections to participate in this international cooperation project. Interested parties should be willing to provide DNA for Type strains in their collections. All microbiologists and institutions from related fields are welcome to submit subprojects for genomic data related research questions. Brief proposals, including questions to be addressed and Type strains to be sequenced and analyzed could be e-mailed to Dr. Juncai Ma at ma@im.ac.cn. Once a proposal is granted as a subproject, the scientist(s) will be asked to lead the full genome analysis and jointly publish the generated outcomes.

## Conclusion:

As a collaborative network of international culture collections, GCM 2.0 will contribute to a genome-based microbial taxonomic framework, establishing high-quality complete genome sequences as the new gold standard. The resulting knowledge and tools generated through this project will not only directly facilitate identifications of

microorganisms, but also improve our ability to predict new genes complexes as well as functions from microbial communities, thus expanding our knowledge of the hitherto undiscovered majority of microbial diversity that may finally lead to sustainable utilization of microbial resources for human benefit.

## List of Abbreviations

| Acronym | Full Name/Institution | Country | Homepage |
|---|---|---|---|
| ATCC | American Type Culture Collection | USA | http://www.atcc.org |
| BCCM | Belgian Coordinated Collections of Microorganisms | Belgium | http://bccm.belspo.be/ |
| CBS | Westerdijk Fungal Biodiversity Institute, formely CBS-KNAW Fungal Biodiversity Centre | Netherlands | http://www.westerdijkinstitute.nl/ |
| CCUG | Culture Collection University of Gothenburg, Sahlgrenska Academy of the University of Gothenburg | Sweden | http://www.ccug.se/ |
| CGMCC | China General Microbiological Culture Collection Center | China | http://www.cgmcc.net |
| CICC | China Center of Industrial Culture Collection | China | http://www.china-cicc.org |
| DSMZ | Leibniz-Institut DSMZ-Deutsche Sammlung von Mikroorganismen und Zellkulturen | Germany | http://www.dsmz.de |
| FGSC | Fungal Genetics Stock Center, Kansas State University, Department of Plant Pathology | USA | http://www.fgsc.net/ |
| JCM | Japan Collection of Microorganisms, RIKEN BioResource Center | Japan | http://jcm.brc.riken.jp/ |
| KCTC | Korean Collection for Type Cultures, Korea Research | Korea | http://kctc.kribb.re.kr/English/inde |

| | | | |
|---|---|---|---|
| | Institute of Bioscience and Biotechnology | | x.aspx |
| MUM | Micoteca da Universidade do Minho, Universidade do Minho | Portugal | http://www.micoteca.deb.uminho.pt |
| NBRC | Biological Resource Center, Department of Biotechnology, National Institute of Technology and Evaluation | Japan | http://www.nite.go.jp/en/nbrc/index.html |
| NCTC | National Collection of Type Cultures, Culture Collections, Public Health England | UK | www.phe-culturecollections.org.uk |
| TBRC | Thailand Bioresource Research Center, National Center for Genetic Engineering and Biotechnology | Thailand | www.tbrcnetwork.org |
| TISTR | TISTR Culture Collection (Bangkok MIRCEN), Thailand Institute of Scientific and Technological Research | Thailand | http://www.tistr.or.th/tistr_culture |
| UCD-FST | Phaff Yeast Culture Collection, Department of Food Science and Technology, University of California Davis | USA | http://phaffcollection.ucdavis.edu/ |
| VKM | All-Russian Collection of Microorganisms, Institute of Biochemistry and Physiology of Microorganisms, Russian Academy of Sciences | Russia | http://www.vkm.ru/ |

## Ethics approval and consent to participate

Not applicable

## Consent for publication

Not applicable

## Availability of data and material

Not applicable


## Acknowledgements

Authors here thank the WFCC Executive Board for supports, and also acknowledge the GCM type strain sequencing project members, partners, and advisors, who have made the cooperation possible. We thank Takashi Gojobori, Guoping Zhao, Xuewei Xu, Xiaoyang Zhi, Hua Xiang, Lei Cai for their insightful suggestions.

## Funding

This work is supported by the Strategic Priority Research Program of the Chinese Academy of Sciences (Grant No. XDA19050301), the Bureau of International Cooperation, Chinese Academy of Sciences (Grant No. 153211KYSB20160029, 153211KYSB20150010), the National key Research Program of China (Grant No 2017YFC1201202, 2016YFC1201303, 2016YFC0901702), the 13th Five-year Informatization Plan of Chinese Academy of Sciences(Grant No. XXH13506), the National Science Foundation for Young Scientists of China(Grant No. 31701157).

## Competing interests

The authors declare that they have no competing interests.

## Authors' contributions

L.H.W drafted the original manuscript text with detailed input from other authors. All authors participated in the GCM type strain sequencing project and have read and approved the final manuscript

## References

1. Curtis TP, Sloan WT, Scannell JW. Estimating prokaryotic diversity and its limits. Proc Natl Acad Sci USA. 2002; 99:10494–9. doi:10.1073/pnas.142680199.

2. Garrity GM. A New Genomics-Driven Taxonomy of Bacteria and Archaea: Are We There Yet? J Clin Microbiol. 2016; 54: 1956-63. doi: 10.1128/JCM.00200-16.

3.Hawksworth DL. The magnitude of fungal diversity: the 1.5 million species estimate revisited. Mycol Res. 2001; 105:1422–1432.

4. Chun J, Oren A, Ventosa A, Christensen H, Arahal DR, da Costa MS, et al. Proposed minimal standards for the use of genome data for the taxonomy of prokaryotes. Int J Syst Evol Microbiol. 2018, 68:461–466. doi: 10.1099/ijsem.0.002516.

5. Kim Y, Koh I, Rho M. Deciphering the human microbiome using next-generation sequencing data and bioinformatics approaches. Methods. 2015; 79-80: 52-9. doi: 10.1016/j.ymeth.2014.10.022.

6. Kang HS. Phylogeny-guided (meta)genome mining approach for the targeted discovery of new microbial natural products. J Ind Microbiol Biotechnol. 2017; 44:285-293. doi: 10.1007/s10295-016-1874-z.

7. Mukherjee S, Seshadri R, Varghese NJ, Eloe-Fadrosh EA, Meier-Kolthoff JP, Göker M, et al. 1,003 reference genomes of bacterial and archaeal isolates expand coverage of the tree of life. Nat Biotechnol. 2017; 35:676–683. doi: 10.1038/nbt.3886.

8. Whitman WB, Woyke T, Klenk HP, Zhou Y, Lilburn TG, Beck BJ, et al. Genomic Encyclopedia of Bacterial and Archaeal Type Strains, Phase III: the genomes of soil and plant-associated and newly described type strains. Stand Genomic Sci. 2015; 10:26. doi: 10.1186/s40793-015-0017-x.

9. 1000 fungal genomes Project. https://jgi.doe.gov/our-science/science-programs/fungal-genomics/1000-fungal-genomes/. Accessed March 1, 2017 .

10. Wu L, Sun Q, Desmeth P, Sugawara H, Xu Z, McCluskey K, et al. World data centre for microorganisms: an information infrastructure to explore and utilize preserved microbial strains worldwide. Nucleic Acids Res. 2017; 45: D611-D618. doi: 10.1093/nar/gkw903.
