# Supplementary material for: The global catalogue of microorganisms 10K type strain sequencing project: closing the genomic gaps for the validly published prokaryotic and fungi species
Source: Gigascience. 2018 Mar 22;7(5):giy026. doi: 10.1093/gigascience/giy026 (PMC5941136; doi:10.1093/gigascience/giy026)
Supplement: Response_to_Reviewer_Comments_Original_Submission.pdf [file giy026_response_to_reviewer_comments_original_submission.pdf]

Author's Response To Reviewer Comments

The full post information and ORCID IDs of the Correspondent authors are provided.

All the revisions suggested by the editors are accepted expect somewhere I added the explanations(highlighted in coloured text in the manuscript).

A version with track changes mode and a clear version were uploaded.
